# The Hypodermic Use of Morphine in the Colic of Horses

**Published:** 1882-10

**Authors:** 


THE HYPODERMIC USE OF MORPHINE IN THE COLIC OF HORSES.—Dr. Carl Lemke, of Bremen, has treated 1,136 cases of colic in horses with hypodermic injections of morphine. He speaks enthusiastically of his success. The morphine must be given in large doses, e. g., seven grains (gr. vii.) for a small horse, eight to ten grains (gr. viii.—gr. x.) for a large one. With these doses he has never seen any injurious effect.

Dr. Lemke acknowledges that morphine is not sufficient in colic from obstruction, but argues that it even assists (?) the action of aloes.

After an injection of morphine, the following three things may happen.

1. The patient after ten or thirty minutes feels no more pain and is well. This happens in the most of his cases.

2. The patient continues better from two to five hours. The pains then return. This shows, says Lemke, that it is a case of obstruction colic. The prognosis is always favorable. One only needs to give purgatives in powerful doses.

3. The patient after the injection shows no, or only very temporary, signs of improvement. These cases usually end fatally.

The hypodermic method helps in the diagnosis and prognosis, therefore Dr. Lemke confesses that much of his practice, though not all, has been in the military service, where he was called to see his cases early.

